# Regional specificity of cortico-thalamic coupling strength and directionality during waxing and waning of spike and wave discharges

**DOI:** 10.1038/s41598-018-37985-7

**Published:** 2019-02-14

**Authors:** Annika Lüttjohann, Hans-Christian Pape

**Affiliations:** Institute of Physiology I, Westfälische Wilhelms-Universität Münster, Münster, Germany

## Abstract

Spike-wave discharges (SWDs) on the EEG during absence epilepsy are waxing and waning stages of corticothalamic hypersynchrony. While the somatosensory cortex contains an epileptic focus, the role of thalamic nuclei in SWD generation is debated. Here we assess the contribution of distinct thalamic nuclei through multiple-site unit recordings in a genetic rat model of absence epilepsy and cross-correlation analysis, revealing coupling strength and directionality of neuronal activity at high temporal resolution. Corticothalamic coupling increased and decreased during waxing and waning of SWD, respectively. A cortical drive on either sensory or higher order thalamic nuclei distinguished between onset and offset of SWD, respectively. Intrathalamic coupling steadily increased during maintained SWD activity, peaked at SWD offset, and subsequently displayed a sharp decline to baseline. The peak in intrathalamic coupling coincided with a sharp increase in coupling strength between reticular thalamic nucleus and somatosensory cortex. This increased influence of the inhibitory reticular thalamic nucleus is suggested to serve as a break for SWD activity. Overall, the data extend the cortical focus theory of absence epilepsy by identifying a regionally specific cortical lead over distinct thalamic nuclei, particularly also during waning of generalized epileptic discharges, thereby revealing a potential window and location for intervention.

## Introduction

The thalamo-cortical system is a complex circuitry within the mammalian nervous system, whose oscillations are crucially implicated in various aspects of sensation, behavior and cognition including the regulation of wakefulness and sleep, attention and the level of consciousness experience^1,2^. Proper regulation of these functions are thought to rely on a balanced interplay of cellular dynamics between the different components of the thalamocortical loops. These regulatory systems govern rhythmic oscillations, while thalamocortical dysrhythmias have been suggested to underlie a multitude of neurological and psychiatric disorders including neuropathic pain, depression, schizophrenia, various sleep disturbances as well as epileptic seizures^3–5^. A prototypical thalamocortical dysrhythmia is childhood absence epilepsy, characterized by spontaneously waxing and waning periods of generalized, hypersynchronous activity. These epileptic seizures can be recorded as spike and wave discharges (SWDs) in the electroencephalogram and induce a loss of conscious experience and responsiveness in the patient. While the existence of a local cortical epileptic focus has been proposed in both humans as well as in genetic rat models of absence epilepsy^6–9^, the contribution of different thalamic nuclei to the generation, maintenance and termination is less well understood. This is especially true for higher order thalamic nuclei, since research on SWD generation and maintenance was mostly focused on first order nuclei^10^. Heterogeneity in intrinsic properties and anatomical connectivity between higher and first order thalamic nuclei, however, may introduce additional dynamics to the system, which might render it vulnerable for the generation of hypersynchronous SWD activity or at the same time might introduce a protective break which ultimately causes the spontaneous termination of an ongoing SWD^10,11^.

A proper understanding of network mechanisms relevant for the generation, maintenance and termination of SWD therefore requires a thorough coupling analysis between cortical focus and both higher and first order thalamic nuclei. The fast and waxing and waning nature of SWD in which neuronal populations are either entrained and synchronized or desynchronized on a millisecond timescale further asks for an analysis, which is able to sketch the fast temporal dynamics within the system.

In the present study, single-unit recordings were simultaneously acquired in deep somatosensory cortex (SCtx) (the proposed epileptic focus) and either first order ventral-postero medial thalamic nucleus (VPM), higher order posterior thalamic nucleus (PO) or reticular thalamic nucleus (RTN) of absence epileptic GAERS rats. SWD on- and offset were analyzed using cross-correlation analysis, assessing dynamic changes in coupling strength and directionality along the transition. Moreover, intrathalamic coupling was assessed from simultaneous recordings of thalamic nuclei, to achieve a detailed and complete picture on both corticothalamic and thalamothalamic coupling dynamics including all relevant areas associated with the assumed focal generation and rapid generalization of SWDs, as well as to unravel network changes relevant for the spontaneous termination of SWDs.

## Material and Methods

### Animals

Thirty-four male GAERS of 3 to 4 months of age were used as experimental subjects.

Animals were born and raised at the Institute of Physiology I, Westfälische Wilhelms-University, Münster, under standard laboratory conditions with a 12-12 h dark-light cycle and *ad libitum* access to water and food.

All experimental procedures were carried out in accordance with the guidelines and regulations of the council of the European Union (Directive 2010/63/EU) and approved by local authorities (review board institution: Landesamt für Natur, Umwelt und Verbraucherschutz Nordrhein-Westfalen; approval ID number: 84-02.04.2014.A398).

### Stereotactic surgery and electrophysiological recordings

Stereotactic surgery and electrophysiological recordings were performed in a stereotactic frame (David Kopf Instruments). For stereotactic surgery rats were anesthetized with pentobarbital (Narcoren, 50 mg/kg IP; Merial GmbH, Münster, Germany), and the local anesthetic Lidocaine was applied to all pressure and incision points. Holes were drilled into the skull for insertion of tungsten electrodes of 1 MΩ (World Precision Instruments, Inc, Sarasota, USA), suited for the combined recording of local field potentials and multi or single unit activity by the same electrode (see below), into two target areas: In a first group of rats (n = 8), electrodes were inserted into the deep layers of the left SCtx (A/P = 0 mm, L = − 4.6 mm, depth between −2.8 and −3.5 mm) and the left PO (A/P = −3.6 mm, L = − 2 mm, depth between −5 and −6 mm); in a second group of rats (n = 7), electrodes were inserted into deep layers of the left SCtx and the left VPM (A/P = −4.16 mm, L = −2.8 mm, depth between −5.2 and −6.1 mm); in a third group of rats (n = 7), electrodes were inserted into deep layers of the left SCtx and the left, caudal RTN (A/P = −2.8, L = −3.75 mm, depth between −5.2 and −6.1 mm); in a fourth group of rats (n = 6), electrodes were inserted into the left RTN and left PO (see above for coordinates), and in a fifth group of rats (n = 6), electrodes were inserted into the left RTN and left VPM (see above for coordinates).

In all rats, two additional holes were drilled into the skull for insertion of a combined ground and reference electrode on top of the cerebellum (right hemisphere) and an epidural LFP recording electrode (both silver wire, diameter 0.25 mm, World Precision Instruments, Inc, Sarasota, USA) on the right somatosensory cortex (A/P = 0 mm, L = +4.6 mm) suited for monitoring purposes. The latter two electrodes were fixed to the skull using dental acrylic cement (Pulpdent Glasslute, Watertown, USA).

Tungsten electrodes were lowered to the region of interest within the target location (see above) and held in place by Direct Drive Micropositioners (David Kopf Instruments) each mounted at an arm of the stereotactic frame. All coordinates were determined relative to bregma according to the stereotactic atlas of Paxinos and Watson^12^.

Single unit activity and local field potential (LFP) recordings were performed under Neurolept anaesthesia/analgesia, as described before by Inoue *et al*.^13^ and Seidenbecher *et al*.^14^. Following stereotactic surgery (see above), Neurolept-anaesthesia/analgesia was induced and maintained via regular i.p injection (every 15–20 min) of the opioid Fentanyl (3.74 mg/kg; Fentanyl; Janssen, Neuss, Germany) and the D2-Antagonist Droperidol (0.033 mg/kg; Xomolix, ProStrakan, Düsseldorf, Germany). The depth of anaesthesia was monitored by breathing rate, epidural LFP recording (potential desynchronization), and responses to noxious stimulation of the hind paw. Upon signs of reduced anesthesia, an additional dose of fentanyl was administered. This anesthesia allows the spontaneous occurrence of SWD in the genetic rat models of absence epilepsy^13–15^.

Signals acquired by a tungsten electrode were preamplified (preamplifier constructed by Electronic Research Group of Medical Faculty, Westfälische Wilhlems University, Münster, Germany) and fed into two ports of an amplifier (DPA-2 FX, NPI Electronics, Göttingen, Germany). Local field potentials and single unit activity were extracted from the signals by filtering between 1 Hz (HP) and 100 Hz (LP) and 500 Hz and 10 kHz, respectively. Local field potentials and unit recordings were subsequently digitzed with a constant sampling rate of 1000 Hz or 20 kHz, respectively, by Cambridge Electronics Data system (CED; 1401plus; Cambridge Electronic Design, Cambridge, UK). This allowed the combined recording of local field potentials and unit activity by the same electrode. Local field potentials acquired via the epidural silver electrode on the right somatosensory cortex were directly fed into the amplifier (DPA-2 FX, NPI Electronics, Göttingen, Germany), filtered between 1 (HP) and 100 (LP) Hz and were digitized at a constant sampling rate of 1000 Hz.

In each recording session, a minimum of 20 SWDs were recorded for a given pair of neurons and a total of two to three pairs of neurons were recorded in each rat. To ensure proper offline sorting of single neuron activity (see below), tungsten electrodes were approached to the neuron until a signal-to-noise ratio of at least twice the background was achieved. The depth of each recorded neurons was registered for subsequent histological verification of the recording site. Each pair of neurons was recorded for an average duration of 1 hour.

### Histology

Following recording of the last pair of neurons within a rat the animal was euthanized by an i.p. injection of 100 mg/kg Pentobarbital (Narcoren; Merial GmbH, Münster, Germany). A direct current was passed through each of the tungsten electrodes to create a micro lesion at the given recording site, which was used for histological verification. The brain was removed from the scull, placed in a 4% paraformaldehyde (PFA) solution for at least 24 h and then fixated in a 30% sucrose solution for another 3 days. Brains were cut into coronal slices of 60 μm, mounted on microscope slides and stained with Cresyl Violet before identification of the location of the micro-lesion with the aid of a light-microscope (Suppl. Fig. 1). Only recordings with histologically verified location within the two target structures (Suppl. Fig. 1) were subject to further analysis.

### Data analysis and statistics

**Figure 1:**
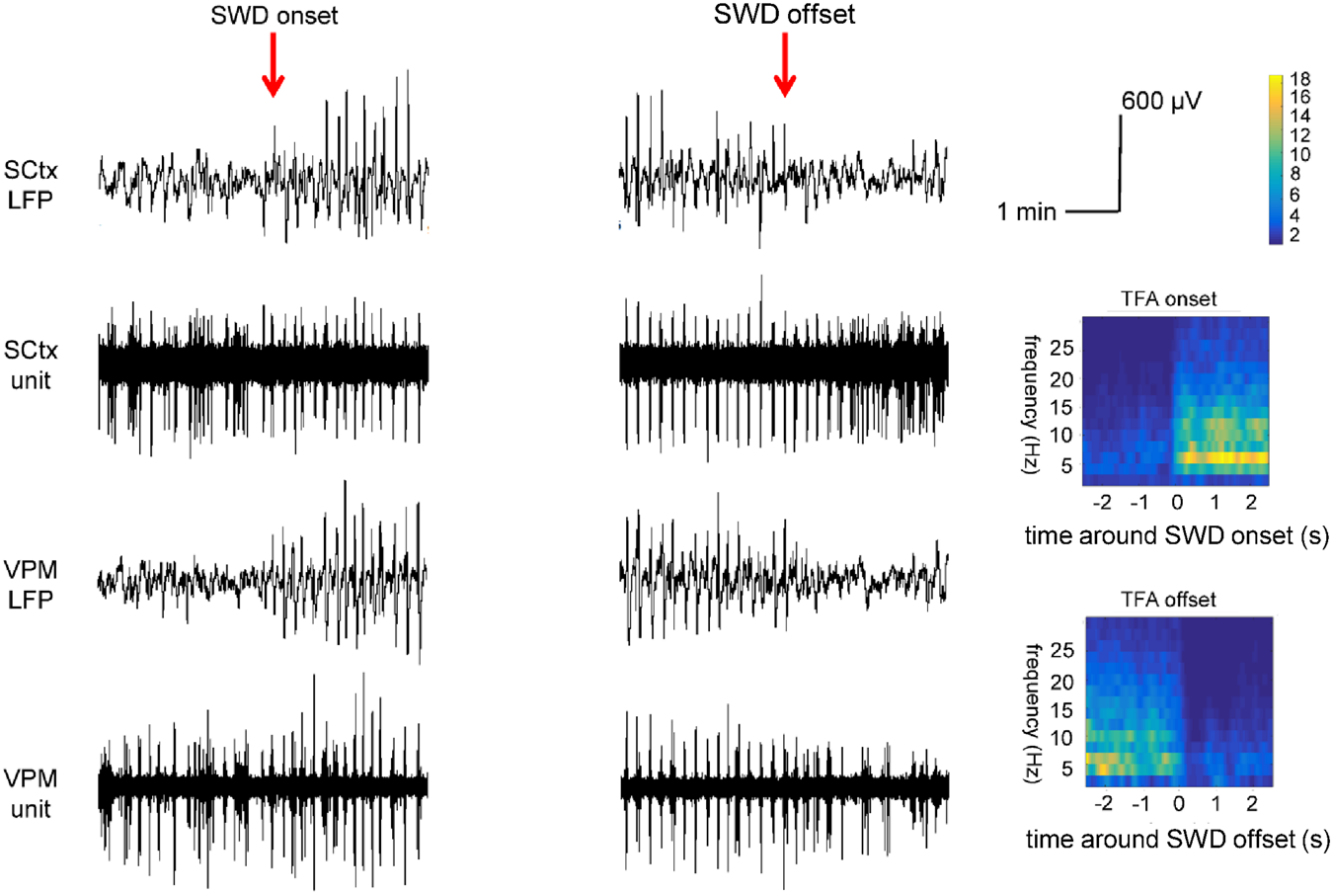
Exemplary recording traces showing simultaneous LFP and unit recordings in SCtx and VPM during six seconds pre-SWD -> SWD (left panel) and SWD -> post-SWD (middle panel) transition periods. Unit activity and LFP signals of the same brain structure were acquired by the same electrode. Red arrows mark the onset and the offset of the SWD respectively. Time frequency analysis (right panel) of the thalamic LFP signal performed during the transition periods. Note the abrupt and strong increase in power (especially at 6 Hz frequency) coinciding with SWD onset and the abrupt and strong decrease in power coinciding with SWD offset.

The onset and offset of SWDs, defined as first or last epileptic spike (sharp spike of at least twice the background LFP), visible in both the cortical and thalamic signals and followed or preceded by rhythmic SWD activity^11,16^, were identified within the LFP recordings by a trained electrophysiologist (Fig. 1). Pre-SWD -> SWD transition periods, displaying LFP and single unit activity starting 3 seconds prior to SWD onset until 3 seconds following SWD onset, as well as SWD -> post SWD transition periods, displaying LFP and single unit activity starting 3 seconds prior to SWD offset until 3 seconds following SWD offset, were selected offline using Spike2 Analysis software.

Unit recordings were subjected to a threshold-based spike sorting procedure in the Spike2 analysis software. Afterwards, LFP and sorted unit recordings of the transition periods were imported into Matlab. Thalamic LFP recordings were subjected to time frequency analysis (TFA) using FieldTrip, a Matlab based open source toolbox for advanced analysis of e.g. electrophysiological data^17^. TFA using Hanning tapering was performed in the frequency range between 2 to 30 Hz: An analysis window of 500 ms was shifted along the transition periods in steps of 50 ms, yielding a frequency resolution of 2 Hz accuracy. Results of TFA served as validation for a correct identification of the time point of SWD on- and offset (Fig. 1). Unit recordings obtained simultaneously in two brain structures (SCtx & PO, SCtx & VPM, SCtx & RTN, RTN & PO, RTN & VPM) were subjected to cross correlation analysis as implemented in Fieldtrip^17^. This analysis reveals the strength as well as the direction of coupling between spike trains at different recording sites by correlating spike trains as a function of timeshift. Coupling strength and directionality were assessed in time windows of 500 ms, sliding along the six seconds lasting transition periods in steps of 125 ms. For the assessment of directionality the analysis window of one signal was shifted back and forth in time relative to the other in steps of 1 ms up to a maximal time shift of 40 ms.

Coupling strength and coupling direction along the pre-SWD -> SWD transition period as well as along the SWD -> post SWD transition was compared between groups of simultaneously recorded neurons/brain structures (SCtx & PO, SCtx & VPM, SCtx & RTN, RTN & PO, RTN & VPM) using a Repeated Measures ANOVA with either coupling strength or coupling direction as dependent variable, timepoint along transition period (from −3 prior to SWD on or offset to +3 s following SWD on or offset) as within subjects factor and group of simultaneously recorded neuron/brain structure (SCtx & PO, SCtx & VPM, SCtx & RTN, RTN & PO, RTN & VPM) as between subject factor.

In order to avoid the multiple comparison problem and reduce the risk of type II errors, post hoc comparisons were performed on 10 selected time bins of interest (t1-t10) along the transition periods, with t1 at baseline (time window −2.625 s and −2.5 before SWD onset), t2 immediately prior to SWD onset (time window −0.375 s and −0.25 s before SWD onset), t3 at SWD onset (time window 0 s and 0.125 s), t4 immediately following SWD onset (time window 0.5 s following SWD onset and 0.625 s following SWD onset), t5 and t6 during stable SWD expression (time window 2.5 s and 2.626 s following SWD onset as well as time window −2.625 s and −2.5 before SWD offset, respectively), t7 immediately prior to SWD offset (time window −0.375 s and −0.25 s before SWD offset), t8 at SWD offset (time window 0 s and 0.125 s), t9 immediatelly following SWD offset (time window 0.5 s and 0.625 s following SWD offset) and t10 at baseline following SWD offset (time window −2.625 s and −2.5 following SWD offset). Values for coupling strength and the direction of coupling of each bin therefore represent average values of two consecutive data points calculated in the cross correlation analysis (see above).

## Results

### Electrophysiological characteristics of SWDs in defined thalamic nuclei and somatosensory cortex

LFP signals of all GAERS recorded under neurolept anesthesia displayed frequent (average 35 per hour) SWDs of 10 to 30 seconds duration at a main frequency of 5–7 Hz (Fig. 1), as observed previously^14,15,18^. During non-epileptic periods, the cortical and thalamic LFP signals were characterized by a dominant theta rhythm, associated with a lightly anaesthetized animal (Suppl. Fig. 2B). Next to the generalized SWDs, the cortical LFP signal also contained periods of short lasting, local epileptic activity (on average 5 per hour) in the form of ‘miniature SWDs’^14^, which were absent in the thalamic traces. These had an average duration of 1 second, often a spindle-like shape but with a clear sharp spike and wave morphology of 6 Hz (Suppl. Fig. 2A). Of note, these local miniature SWDs in the cortical LFP coincided with local spike locked unit activity recorded in SCtx, while no such local epileptic miniature SWDs were found in any of the thalamic traces (χ^2^_(number of local SWD in cortex vs number of local SWD in thalamus)_: p < 0.001) (Suppl. Fig. 2A).

Associated neuronal activity patterns depicted in single unit recordings acquired by the same electrode characteristically differed in the four recorded structures. During SWDs, cortex and all three thalamic nuclei showed rhythmic bursting activity phase-locked to the LFP spike of the seizure (Fig. 1, Suppl. Fig. 3). Typically, bursts recorded in the RTN had a longer duration of approximately 30.7 ms as compared to VPM (17.9 ms), PO (12.5 ms) and somatosensory cortex (16.8 ms) (Suppl. Fig. 3).

During nonepileptic periods, the cortex, PO and VPM showed a mixture of single spikes and interspersed burst activity with a slightly higher burst activity/frequency seen for VPM and PO neurons as compared to neurons recorded in the somatosensory cortex (~8 vs. 11 burst within 5 sec). Towards the onset of the SWD bursting activity tended to become more frequent in all three structures (~20 burst within 5 sec).

For RTN neurons, the nonepileptic period was characterized by pronounced bursting activity, which did not show any obvious change in frequency towards the beginning of the SWD (~19 bursts within 5 sec). However, immediately following SWD offset, the RTN displayed a slightly increased inter-burst interval (~2–3 bursts within 1 sec), while the SCtx often demonstrated a short period of regular, high frequent, tonic firing activity (Fig. 4A).

### Cortico-thalamic dynamics in coupling strength and coupling direction

In order to relate the observed firing patterns to coupling dynamics, relevant for the generation and termination of SWD, unit recordings were subject to cross correlation analysis. Cross correlation analysis determines the strength of coupling as well as the direction of coupling (i.e. the direction of information transfer, either cortex leading thalamus or thalamus leading cortex or bidirectional coupling). To this end, an analysis window of 500 ms was shifted in steps of 125 ms along the pre-SWD->post SWD transition period, displaying unit activity recorded 3 sec prior to SWD onset until 3 sec following SWD onset. Similarly the analysis window was shifted along the SWD->post SWD transition period, displaying unit activity recorded 3 sec prior to SWD offset until 3 sec following SWD offset. At each of the time steps along the transition periods, coupling strength and coupling direction were assessed in order to detect the temporal dynamics of changes in coupling strength and coupling direction.

**Figure 2:**
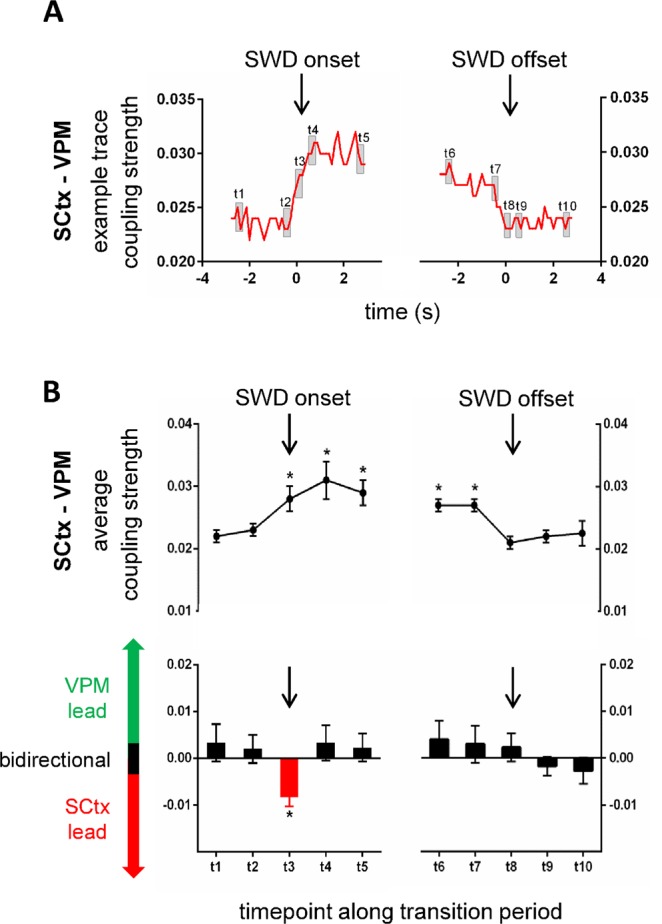
Coupling dynamics between SCtx and VPM along transition periods characterizing SWD on- and offset. (**A**) Exemplary coupling strength between SCtx and VPM along the pre-SWD -> SWD (left) and the SWD -> post-SWD (right) transition period. Black arrows indicate the timepoint of SWD onset and SWD offset, respectively. Grey squares indicate time intervals of interest, which were used for in depth statistical analysis (see below) (**B**) (upper panel): Average coupling strength along the pre-SWD -> SWD and SWD -> post-SWD transition periods represented for 10 time-intervals of interest including t1 at baseline (time window −2.625 s and −2.5 before SWD onset), t2 immediately prior to SWD onset (time window −0.375 s and −0.25 s before SWD onset), t3 at SWD onset (time window 0 s and 0.125 s), t4 immediately following SWD onset (time window 0.5 s following SWD onset and 0.625 s following SWD onset), t5 and t6 during stable SWD expression (time window 2.5 s and 2.626 s following SWD onset as well as time window −2.625 s and −2.5 before SWD offset, respectively), t7 immediately prior to SWD offset (time window −0.375 s and −0.25 s before SWD offset), t8 at SWD offset (time window 0 s and 0.125 s), t9 immediately following SWD offset (time window 0.5 s and 0.625 s following SWD offset) and t10 at baseline following SWD offset (time window −2.625 s and −2.5 following SWD offset). (**B**) (lower panel): Average coupling direction along the pre-SWD -> SWD and SWD -> post-SWD transition periods represented for 10 time-intervals of interest (see B upper panel for details). Black bars indicate a bidirectional coupling between the two brain structures; red bars indicate a unidirectional cortical lead, and green bars indicate a unidirectional thalamic lead. Note the absence of VPM lead and the unidirectional cortical lead at SWD onset (t3).

**Figure 3:**
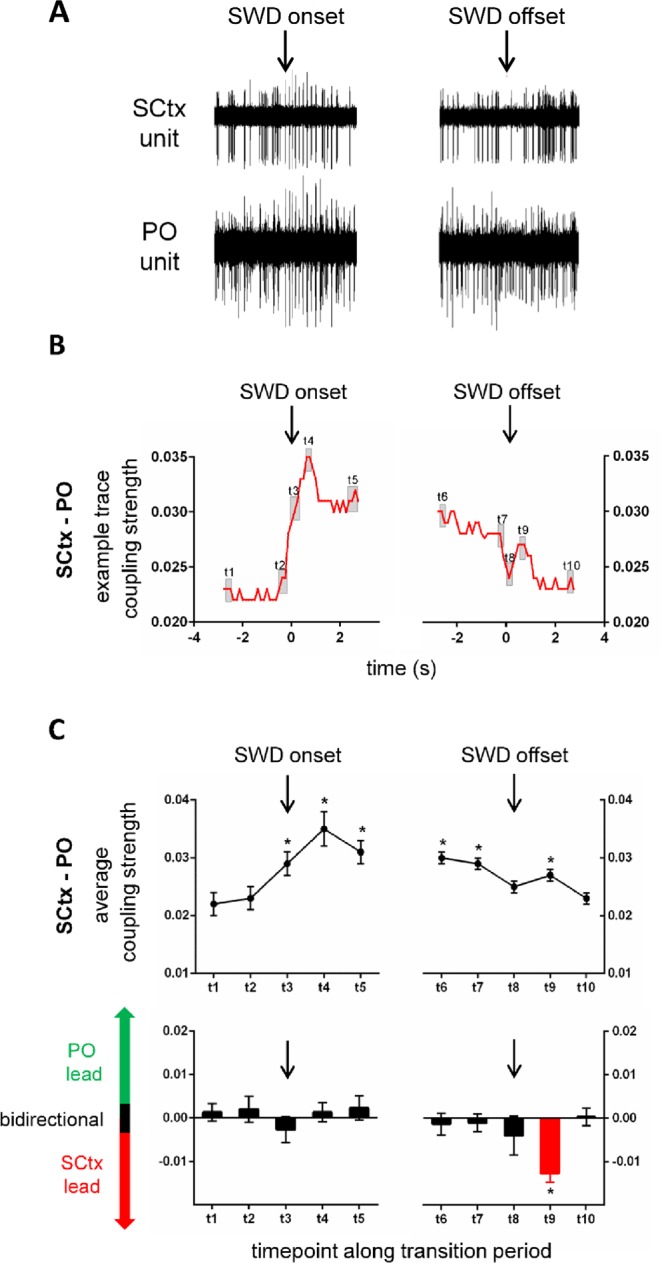
Coupling dynamics between SCtx and PO along transition periods characterizing SWD on- and offset. Exemplary recording traces of unit activity in SCtx and PO (**A**) and coupling strength (**B**) between SCtx and PO along the pre-SWD -> SWD (left) and the SWD -> post-SWD (right) transition period. Black arrows indicate the timepoint of SWD onset and SWD offset respectively. Grey squares indicate time-intervals of interest, which were used for in depth statistical analysis (see below). (**C**) (upper panel): Average coupling strength along the pre-SWD -> SWD and SWD -> post-SWD transition periods represented for 10 time-intervals of interest including t1 at baseline (time window −2.625 s and −2.5 before SWD onset), t2 immediately prior to SWD onset (time window −0.375 s and −0.25 s before SWD onset), t3 at SWD onset (time window 0 s and 0.125 s), t4 immediately following SWD onset (time window 0.5 s following SWD onset and 0.625 s following SWD onset), t5 and t6 during stable SWD expression (time window 2.5 s and 2.626 s following SWD onset as well as time window −2.625 s and −2.5 before SWD offset, respectively), t7 immediately prior to SWD offset (time window −0.375 s and −0.25 s before SWD offset), t8 at SWD offset (time window 0 s and 0.125 s), t9 immediately following SWD offset (time window 0.5 s and 0.625 s following SWD offset) and t10 at baseline following SWD offset (time window −2.625 s and −2.5 following SWD offset). (**C**) (lower panel): Average coupling direction along the pre-SWD -> SWD and SWD -> post-SWD transition periods represented for 10 time-intervals of interest (see C upper panel for details). Black bars indicate a bidirectional coupling between the two brain structures; red bars indicate a unidirectional cortical lead and green bars indicate a unidirectional thalamic lead. Note the absence of PO lead,  the bidirectional coupling at SWD onset (t3), and the cortical lead immediately following SWD offset (t9).

**Figure 4:**
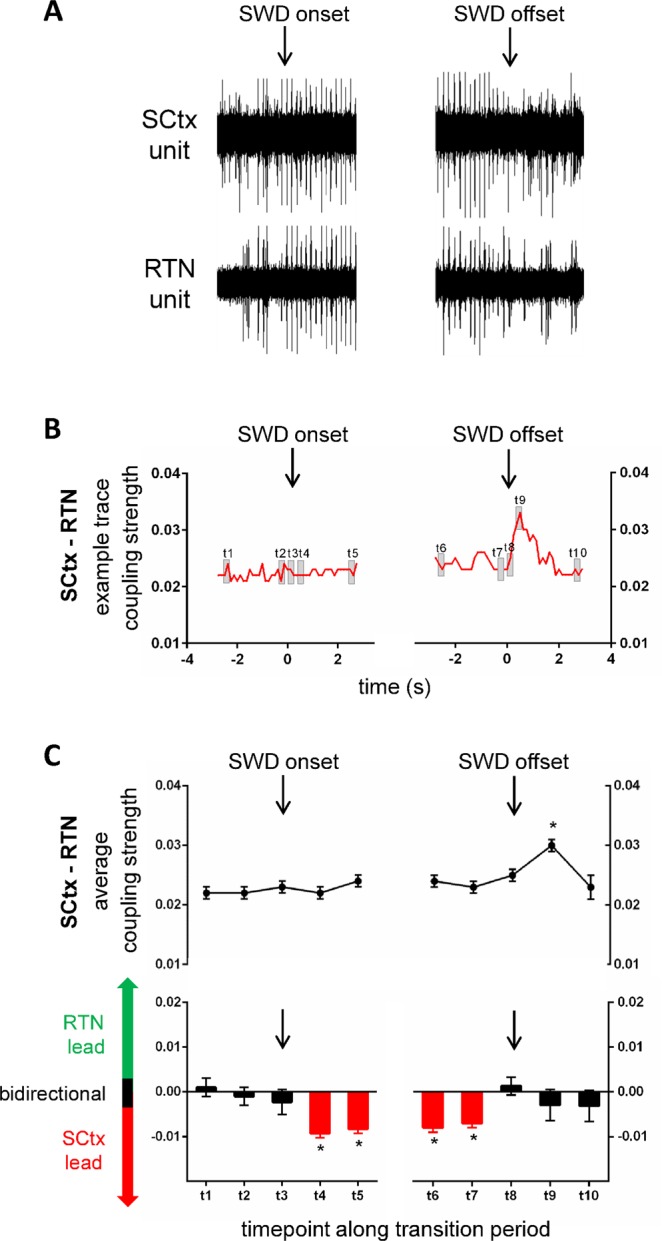
Coupling dynamics between SCtx and RTN along transition periods characterizing SWD on- and offset. Exemplary recording traces of unit activity in SCtx and RTN (**A**) and coupling strength (**B**) between SCtx and RTN along the pre-SWD -> SWD (left) and the SWD -> post-SWD (right) transition period. Black arrows indicate the timepoint of SWD onset and SWD offset respectively. Grey squares indicate time-intervals of interest, which were used for in depth statistical analysis (see below). (**C**) (upper panel): Average coupling strength along the pre-SWD -> SWD and SWD -> post-SWD transition periods represented for 10 time-intervals of interest including t1 at baseline (time window −2.625 s and −2.5 before SWD onset), t2 immediately prior to SWD onset (time window −0.375 s and −0.25 s before SWD onset), t3 at SWD onset (time window 0 s and 0.125 s), t4 immediately following SWD onset (time window 0.5 s following SWD onset and 0.625 s following SWD onset), t5 and t6 during stable SWD expression (time window 2.5 s and 2.626 s following SWD onset as well as time window −2.625 s and −2.5 before SWD offset, respectively), t7 immediately prior to SWD offset (time window −0.375 s and −0.25 s before SWD offset), t8 at SWD offset (time window 0 s and 0.125 s), t9 immediately following SWD offset (time window 0.5 s and 0.625 s following SWD offset) and t10 at baseline following SWD offset (time window −2.625 s and −2.5 following SWD offset).Note the strong short lasting increase in coupling strength immediately following SWD offset (t9). C (lower panel): Average coupling direction along the pre-SWD -> SWD and SWD -> post-SWD transition periods represented for 10 time-intervals of interest (see C upper panel for details). Black bars indicate a bidirectional coupling between the two brain structures; red bars indicate a unidirectional cortical lead and green bars indicate a unidirectional thalamic lead. Note the persistent cortical lead during the SWD.

Changes in coupling strength and coupling direction along the pre-SWD -> SWD and along the SWD->post-SWD transition period were characteristically different for the three corticothalamic groups of simultaneously recorded neurons/brain structures (SCtx-VPM, SCtx-PO, SCtx-RTN) (Figs 2A, 3B and 4B) (Repeated Measures ANOVA_couping strength_pre-SWD->SWD transition_: group × time interaction: F(184, 3174) = 2.326, p < 0.005; Repeated Measures ANOVA_couping direction_pre-SWD->SWD transition_: group × time interaction: F(184, 3174) = 1.426, p < 0.05; Repeated Measures ANOVA_couping strength_SWD->post-SWD transition_: group × time interaction: F(184, 3174) = 1.823, p < 0.005; Repeated Measures ANOVA_couping direction_SWD->post-SWD transition_: group × time interaction: F(184, 3174) = 1.475, p < 0.05).

To further disentangle these differences, coupling strength and coupling direction observed during a baseline period t1 (assessed at −2.625 s and −2.5 before SWD onset) was compared to coupling strength and coupling direction assessed during 9 time intervals of interest. More specifically, t2 immediately prior to SWD onset (time window −0.375 s and −0.25 s before SWD onset), t3 at SWD onset (time window 0 s and 0.125 s), t4 following SWD onset (time window 0.5 s following SWD onset and 0.625 s following SWD onset), t5 and t6 during stable SWD expression (time window 2.5 s and 2.626 s following SWD onset as well as time window −2.625 s and −2.5 before SWD offset, respectively), t7 immediately prior to SWD offset (time window −0.375 s and −0.25 s before SWD offset), t8 at SWD offset (time window 0 s and 0.125 s), t9 shortly following SWD offset (time window 0.5 s and 0.625 s following SWD offset) and t10 at baseline following SWD offset (time window −2.625 s and −2.5 following SWD offset). These time intervals were separately assessed for each of the three corticothalamic groups of simultaneously recorded neurons.

Activity in SCtx – VPM displayed a coupling strength of 0.022 during baseline (t1) and remained on this level immediately prior to SWD onset (t2 = 0.023) (p > 0.05). At SWD onset, coupling strength significantly increased to 0.028 (t3) (p < 0.05) and remained on this increased level immediately following SWD onset (t4 = 0.031) (p < 0.05), during stable SWD expression (t5 = 0.029, t6 = 0.027) (both p < 0.05) and immediately prior to SWD offset (t7 = 0.027) (p < 0.05). At SWD offset coupling strength between SCtx and VPM returned back to baseline coupling values (t8 = 0.021) (p > 0.05) and remained on this degree of coupling immediately following SWD offset (t9 = 0.022) (p > 0.05) as well as at baseline following SWD offset (i.e. 2.625 s and 2.5 following SWD offset) (p > 0.05) (Fig. 2B, upper trace).

The associated coupling direction between SCtx and VPM was found to be bidirectional during the baseline period (t1) as well as immediately prior to SWD onset (t2) (p > 0.05). Coupling switched to unidirectionality showing a dominant cortical lead onto the VPM at SWD onset (t3) (p < 0.05) and returned to bidirectionality following SWD onset (t4) (p > 0.05). Coupling direction between SCtx and VPM remained bidirectional during stable SWD expression (t5, t6) (both p > 0.05), immediately prior to SWD offset (t7) (p > 0.05) at SWD offset (t8) (p > 0.05), shortly following SWD offset (t9) (p > 0.05) and at baseline following SWD offset (i.e. −2.625 s and −2.5 following SWD offset) (p > 0.05) (Fig. 2B, lower panel).

Activity in SCtx and PO showed a baseline coupling strength of 0.022 (t1), followed by a significant increase in coupling strength at SWD onset (t3 = 0.029) (p < 0.05). Coupling remained increased during the SWD (t4 = 0.035, t5 = 0.031, t6 = 0.030, t7 = 0.029) (all p < 0.05) and returned to baseline strength at SWD offset (t8 = 0.025) (p > 0.05). However, immediately following SWD offset (t9) a short lasting significant increase in coupling strength between SCtx and PO was noticed (t9 = 0.027) (p < 0.05), while coupling strength was at baseline values thereafter (t10 = 0.023) (p > 0.05) (Fig. 3C, upper trace).

The associated coupling direction between SCtx and PO was bidirectional during the baseline period (t1) and the complete SWD (t3–t8, all p > 0.05). A significant change towards a unidirectional coupling with SCtx leading the PO occurred immediatelly following SWD offset (t9, p < 0.05). This unidirectional cortical lead onto the PO returned to a bidirectional coupling between SCtx and PO thereafter (t10, p > 0.05). (Fig. 3C, lower trace).

Activity in SCtx – RTN showed a coupling strength of 0.022 during the baseline period (t1) and did not significantly change at SWD onset and during the SWD (t3–t8 (all p > 0.05). However, a strong and short lasting increase in coupling strength to 0.030 occurred immediately following SWD offset (t9, p < 0.05), while coupling strength returned to baseline value thereafter (t10 = 0.023) (p > 0.05) (Fig. 4C, upper trace). The coupling direction between SCtx and RTN was bidirectional at baseline (t1), and changed towards a unidirectional cortical lead onto the RTN immediately following SWD onset (t4) (p < 0.05). This unidirectional coupling with the cortex leading the RTN persisted during the SWD (t5, t6, t7, all p < 0.05) and returned to a bidirectional coupling at SWD offset (t8, t9, t10; all p > 0.05) (Fig. 4C, lower trace).

### Intrathalamic dynamics in coupling strength and coupling direction

Since the RTN receives direct input by SCtx via collaterals of cortico-thalamic neurons to the VPM, but does not directly connect to SCtx, and since intrathalamic mechanisms significantly contribute to SWDs^19^, coupling dynamics between RTN and recorded thalamic nuclei were analyzed next.

Coupling dynamics during the pre-SWD-> SWD and during the SWD-> post-SWD transition period were similar between RTN-PO and RTN-VPM, both in terms of coupling strength and coupling directionality (Repeated Measures ANOVA_couping strength_: group × time interaction: F(9, 198) = 0.76, p > 0.05; Repeated Measures ANOVA_couping direction_: group × time interaction: F(9, 198) = 0.538, p > 0.05). RTN and PO as well as RTN and VPM showed a significant gradual increase in coupling strength during the SWD that reached a maximum at t9 (immediately following SWD offset) (Repeated Measures ANOVA_couping strength_: main effect of time: F(9, 198) = 5.969, p < 0.001; t3 to t9, all p < 0.05) (Suppl. Fig. 4, lower trace). Coupling direction remained bidirectional between RTN and PO as well as RTN and VPM during the complete pre-SWD-> SWD and SWD -> post-SWD transition period (Repeated Measures ANOVA_couping direction_: main effect of time: F(9, 198) = 0.768, p > 0.05; t1 to t10, all p > 0.05) (data not shown).

## Discussion

The current study investigated coupling dynamics associated with the generation and termination of SWDs within the thalamocortical system of absence epileptic GAERS. Coupling strength and coupling directionality were assessed from single unit recordings obtained in anaesthetized GAERS during waxing and waning periods of spontaneously occurring SWDs for five groups of simultaneously recorded neurons/brain structures (SCtx-VPM, SCtx-PO, SCtx-RTN, RTN-VPM and RTN-PO), deciphering coupling dynamics between multiple relevant areas associated with the assumed focal generation and rapid generalization of SWDs. Most of these groups of simultaneously recorded neurons/brain structures (SCtx-VPM, SCtx-PO, RTN-VPM, RTN-PO) showed a fast increase in coupling strength at SWD onset. This increased coupling strength was maintained during the SWD and quickly returned to baseline values at SWD offset.

However, within this scheme, regionally specific coupling dynamics were noticed:

1. Coupling strength did not significantly change at SWD onset between SCtx and RTN, in contrast to all other recorded brain regions (SCtx-VPM, SCtx- PO, RTN-PO, RTN-VPM).
2. Coupling was bidirectional at baseline, and radically changed at SWD onset to unidirectional SCtx lead on VPM and RTN. Notably, the change in directionality persisted throughout the SWD in SCtx -> RTN, was restricted to the very onset of the SWD in SCtx -> VPM, and missing in SCtx-PO.
3. At SWD offset, coupling strength and directionality returned to baseline in all recorded regions, with two notable exceptions. First, SCtx -> PO changed to unidirectional cortical lead accompanied by an increase in coupling strength (seen during time interval t9). Second, coupling strength between SCtx and RTN showed a sharp and short lasting increase (seen during time interval t9).
4. Compared to observed changes in thalamocortical activity, coupling strength within the thalamus (RTN-VPM and RTN-PO) showed a more gradual increase during the SWD. Of note, the maximal increase in coupling strength between RTN-VPM and RTN-PO was reached during time interval t9 (i.e. immediately following SWD offset).

Coupling dynamics and directionality in neuronal constituents of a distributed synaptic network are a prerequisite for understanding maladapted transformation of network activity, as seen for the thalamo-cortical dysrhythmias during absence epilepsy. In the present study, coupling dynamics were assessed with the aid of cross correlation analysis, an amplitude-based method which determines the degree of coupling between two signals as a function of time shift. The introduction of the time shift allows to determine the direction of coupling between signals which can help to identify and to infer interdependencies between brain structures and information flow within the system (e.g. where does a seizure start, how does another structure respond, to which structures is SWD activity transmitted)^20^. While cross-correlation analysis can be applied to local field potential data, analyses of unit activity, as performed in the current study, represents a more direct measurement of local neuronal activity, reflecting brain dynamics with no influence of confounding factors like for example volume conduction^20,21^. It is thus a reliable and versatile method to study (fast) network dynamics, and can be regarded as one of the most direct assessments for cellular network dynamics^20^. In addition to that, recording and analysis of unit activity in the anaesthetized rat, as performed in the current study, enables stable high quality recordings with appropriate signal-to-noise ratio and ensures that observed changes in coupling are exclusively related to mechanisms of SWD generation and termination and not to changes in for example the level of vigilance, often seen to co-occur with SWD generation in freely moving animals. SWD in GAERS show a main frequency of 6–8 Hz, which is slightly higher than the SWD frequency of 2–4 Hz seen in children^22,23^. The difference is, amongst others, attributed to a difference in GABAergic conductance profiles within the cortico-thalamo-cortical system, with GABA_A_ receptor-mediated dominance in the rat models and a major contribution of GABA_B_ receptors in children^24^.

The above outlined regionally specific changes demonstrate the heterogeneity of coupling dynamics within the thalamocortical system and indicate differences in the functional contribution of the network structures for the generation and termination of SWD. In the established models and theories on the generation of SWDs, the somatosensory cortex is proposed to contain a local, hyperexcitable seizure onset zone^25,26^. Intracellular recordings from layer 5 and 6 of this area reveal a strong increase in action potential firing shortly prior to SWD onset^7,27^. Moreover, LFP recordings, obtained at multiple cortical and thalamic sites, analyzed with a non-linear association analysis, revealed that the somatosensory cortex leads all other cortical and thalamic sites during the first 500 ms of the SWD. Thereafter, cortex and thalamus were found to lead each other (i.e. to keep a bidirectional coupling) to maintain the SWD^6,16^. The increase in coupling strength between SCtx – VPM and especially the unidirectional cortical lead onto the VPM at SWD onset, revealed within the single unit recordings acquired in the current study, are in full agreement with this established theory on SWD generation. It support the hypothesis of an epileptic onset zone in SCtx, combining both the accuracy of unit recordings as well as the valuable information of coupling directionality. Furthermore, local miniature SWD, as earlier reported by Seidenbecher and colleagues^14^ to occur in LFP recordings of the SCtx but not in the thalamus, were found to coincide with local, spike-locked burst firing of neurons in the deep SCtx, with no concomitant unit activity in thalamic recordings. These findings confirm the local cortical origin of these miniature SWD and further validate the cortical focus theory of SWD generation.

A change towards a unidirectional cortical drive at SWD onset, supporting the cortical focus theory was also seen between SCtx and RTN. This unidirectional influence is likely to be transmitted via the collaterals of cortico-thalamic axons from cortical layer 6 to VPM and RTN. Despite a somewhat surprising absence of coupling strength increase, this persistent unidirectional cortical lead (influence) on the RTN is tentatively proposed to result in the observed intrathalamic changes in coupling. In other words, the persistent activation of the GABAergic RTN by corticothalamic collaterals in turn results into the gradual and persistent increase in coupling strength between RTN and VPM. Similar considerations hold for RTN and PO, via the GABAergic projections of the RTN on thalamocortical neurons of VPM and PO. In this way, the RTN is suggested to function as a synchronizer of intrathalamic and thalamo-cortical activity proposed to be crucial for the generation and maintanace of SWD by the currently established models and theories on SWD generation. The RTN slightly hyperpolarizes thalamocortical neurons and promotes the rhythmic burst firing, mediated via low threshold Ca^2+^ spikes, during the SWD^15,18,19,22,28^.

One notable exception form the simple cortical to thalamus lead at SWD onset was the PO nucleus, which showed an increase in *bidirectional* coupling with the SCtx during the first 500 ms of the SWD. The PO therefore is the only thalamic nucleus, recorded in this study, which can provide a resonance loop for SWD activity within its onset period. In fact, such a feedback was suggested to be crucial for the generation of network oscillations like SWDs^16,29,30^ and therefore highlight the importance of the PO for SWD generation. The PO nucleus is a higher order thalamic nucleus, reciprocally connected to SCtx. It receives input via glutamatergic excitatory corticofugal axons in layer 5, which, unlike corticothalamic axons to the VPM, do not give rise to collaterals to the RTN^31,32^. Given this difference as well as different intrinsic properties^33–35^, higher order nuclei have recently been proposed to add additional yet unexplored dynamics to a system, which might render it prone for the generation of hypersynchronous activity^10,36^. The unique bidirectional coupling of the higher order PO with the SCtx at SWD onset can be seen as support for this suggestion, and completes an important aspect to our understanding on how the thalamocortical system, is rendered prone for the generation of hypersynchronous activity.

In contrast to SWD generation, for which models and theories have been proposed in literature, little investigation has taken place concerning network mechanisms relevant for SWD termination. In the current study three outstanding changes occurring time-locked to each other during time-interval t9 (i.e. immediately following SWD offset (time window 0.5 s and 0.625 s following SWD offset)) were revealed within the paired single unit recordings of GAERS, including: 1. Coupling strength between SCtx and RTN showed a sharp and short lasting increase; 2. The gradual increase in coupling strength between RTN-VPM and RTN-PO seen during seen SWD reached its maximum; and 3. SCtx -> PO changed for the first time to a unidirectional cortical lead accompanied by an increase in coupling strength.

As was the case for SWD generation, these region-specific changes demonstrate the heterogeneity of coupling dynamics within the thalamocortical system. Furthermore, compared to the changes at SWD onset, described and discussed above, it also highlights that SWD termination constitutes more than a simple reversion of the changes seen with SWD onset.

In accordance with the above line of reasoning considering the coupling changes between SCtx and RTN as well as between RTN - VPM and RTN – PO at SWD onset, it is proposed that the persistent cortical lead onto the RTN seen during the SWD results in a persistent activation of GABAergic neurons of the RTN. The RTN neurons project onto the thalamic nuclei and in turn mediate a gradual increase in coupling strength between RTN and PO as well as RTN and VPM, reaching its maximum during time interval t9 (immediately following SWD termination). It is tentatively proposed that the sharp short lasting increase in coupling strength at t9 between SCtx and RTN reflects this maximal intrathalamic coupling synchrony. It is interesting to consider the existence of a threshold of inhibitory coupling of the RTN onto the thalamic nuclei, at which the RTN changes from a ‘synchronizer’ towards a ‘gate keeper’ that ultimately inhibits excessive thalamocortical activity and prevents SWD activity from being continued. A gate keeper function of the RTN has already been mentioned in the framework of sleep-wake regulation and selective attention^37–39^. In support for the ‘gate keeper’ role of the RTN that supports termination of the SWD, it has been shown that local pharmacological application of GABA_B_ receptor antagonists, promoting RTN burst firing^40^, in the RTN of absence epileptic rats significantly reduced SWD activity^41,42^. By contrast, lesions to the caudal RTN resulted in an increase in SWD activity^43^.

Finally, the radical shift from bidirectional to unidirectional cortical lead between SCtx and PO during time-inteval t9 is suggestive of the strong inhibition provided by the RTN, revealing a reduced feedback of the PO to the SCtx and resulting discontinuation of the network oscillation (see also^11^. This change can therefore be regarded as another supporting evidence for the importance of PO feedback for the generation of SWD.

In sum, by deciphering corticothalamic network dynamics with an analysis that combines the unbiased accuracy of unit recordings as well as the valuable information of coupling directionality, the present study directly validates the established models on SWD generation. Moreover, the present study extends these models by revealing the importance of the PO for SWD generation as well as the importance of the RTN for SWD termination. Next to the role of a synchronizer during SWD, the RTN likely takes over the function of a gate keeper, dampening hypersynchronous cortico-thalamic activity and finally terminating the SWD. Lastly, this study demonstrates the heterogeneity of network dynamics for different thalamic nuclei on a pathophysiological level. Similar differences in network dynamics might be expected on a physiological level for the regulation of thalamocortical oscillations relevant for various cognitive processes^37^.

## Supplementary information

Supplementary Information
