# Supplementary material for: Regional specificity of cortico-thalamic coupling strength and directionality during waxing and waning of spike and wave discharges
Source: Sci Rep. 2019 Feb 14;9:2100. doi: 10.1038/s41598-018-37985-7 (PMC6375974; doi:10.1038/s41598-018-37985-7)
Supplement: Supplementary file 1 — Supplementary Information [file 41598_2018_37985_MOESM1_ESM.docx]

**Regional specificity of cortico-thalamic coupling strength and directionality during waxing and waning of spike and wave discharges**

Annika Lüttjohann and Hans-Christian Pape

| 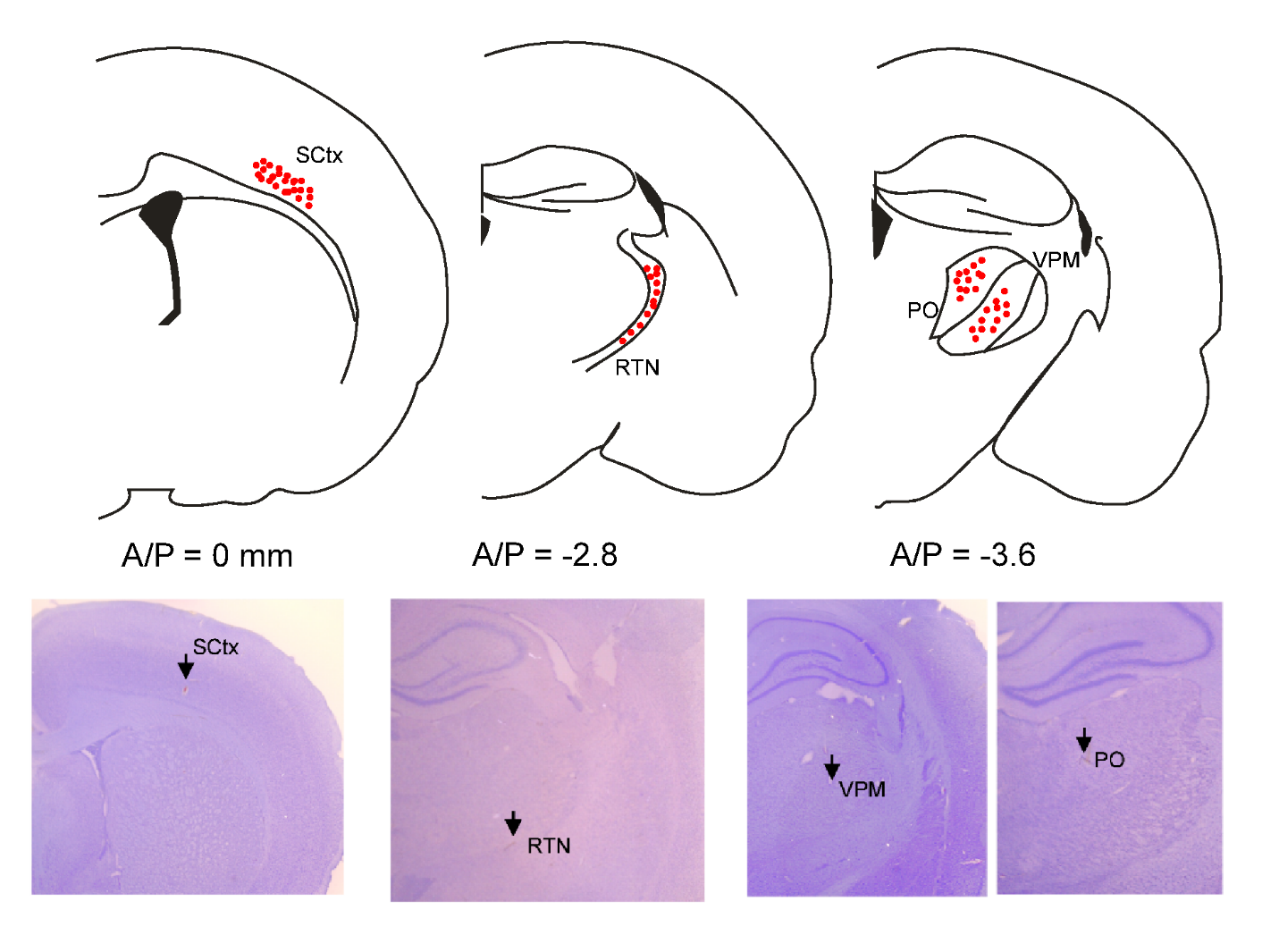 |
| --- |
| **Suppl. Fig. 1** Histological verification of recording sites within the four target structures. Upper diagrams are schematic representations of SCtx (left) at A/P = 0 mm relative to Bregma, RTN (middle) at A/P = -2.8 mm relative to Bregma as well as VPM and PO (right) displayed at A/P = -3.6 mm relative to Bregma. Lower figures display examples of lesions (arrowheads) in cresyl violet stainings at sites indicated. |

| 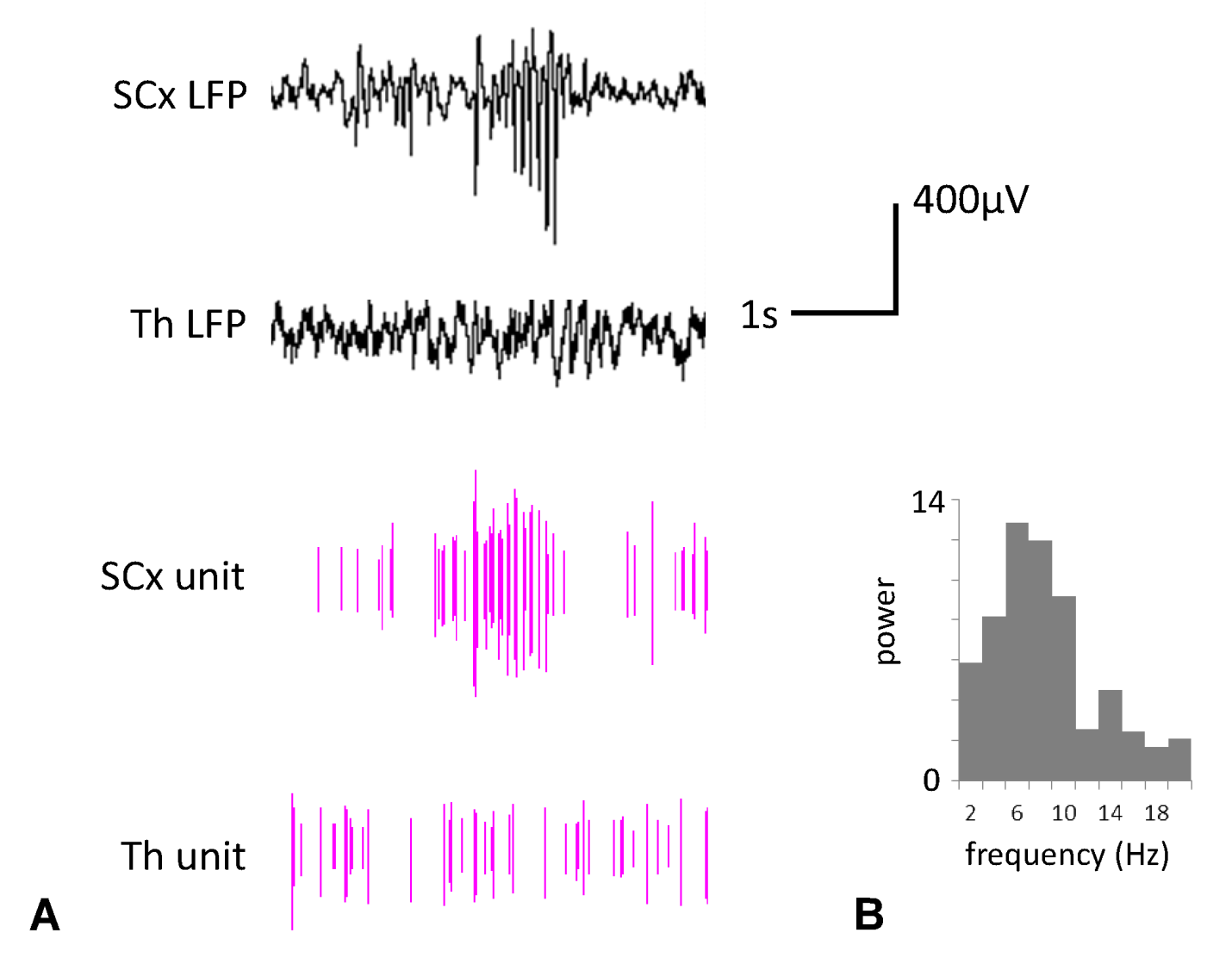 |
| --- |
| **Suppl. Fig 2** A: Exemplary recording of a local ‘miniature SWD’. Note that these miniature SWD are only present in the cortical LFP recording concurrent with spike-locked unit activity of the cortical neuron in SCtx, while no such activity is present in the thalamic traces. B: Power spectrum of the interictal cortical LFP. Note the dominant theta power at 6-8 Hz characteristic for a lightly anaesthetized animal. |

| 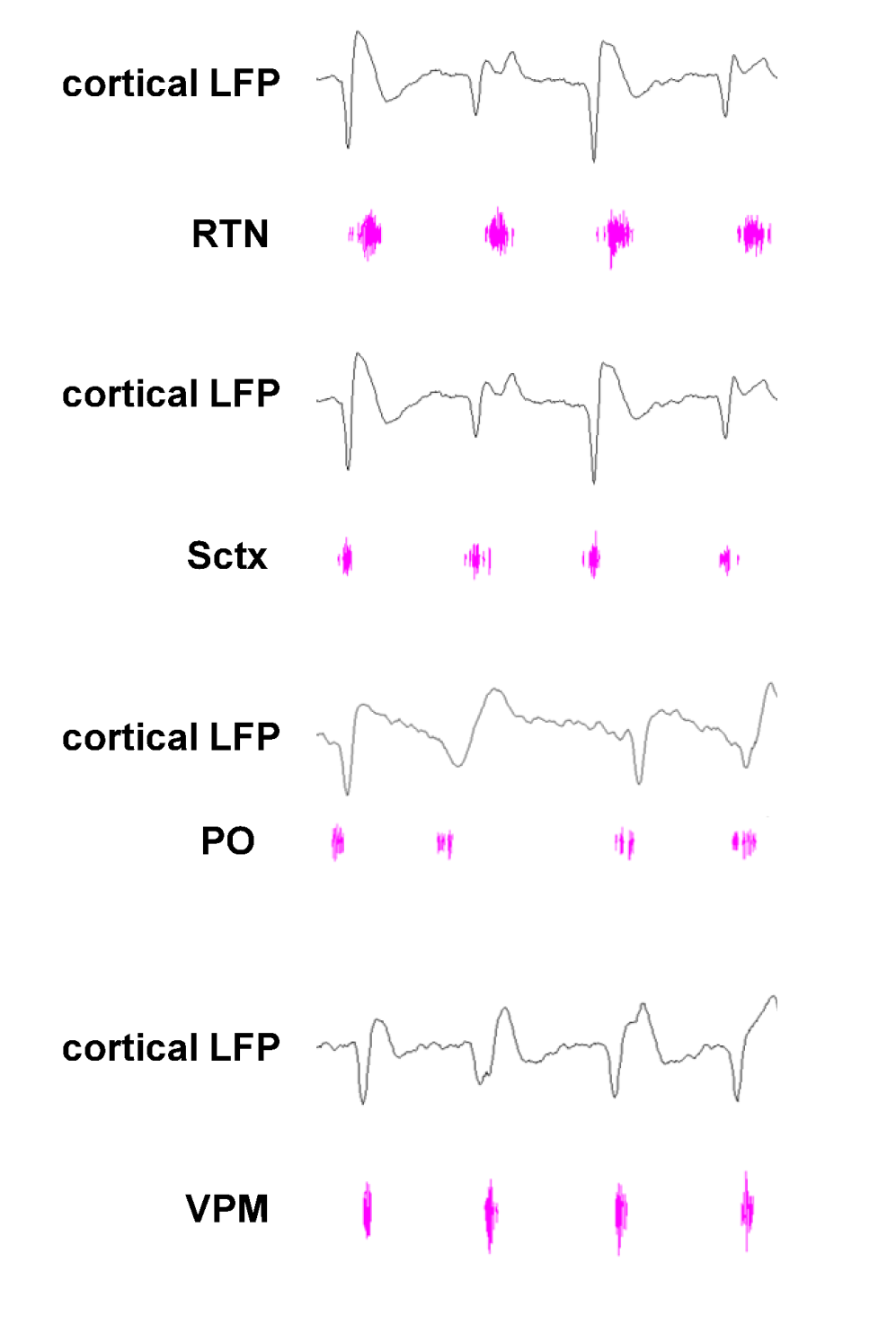 |
| --- |
| **Suppl. Fig. 3** Rhythmic burst firing of neurons in RTN, SCtx, PO and VPM during the SWD. Note the slightly longer burst duration of the RTN neuron as compared to SCtx, VPM and PO. |

| 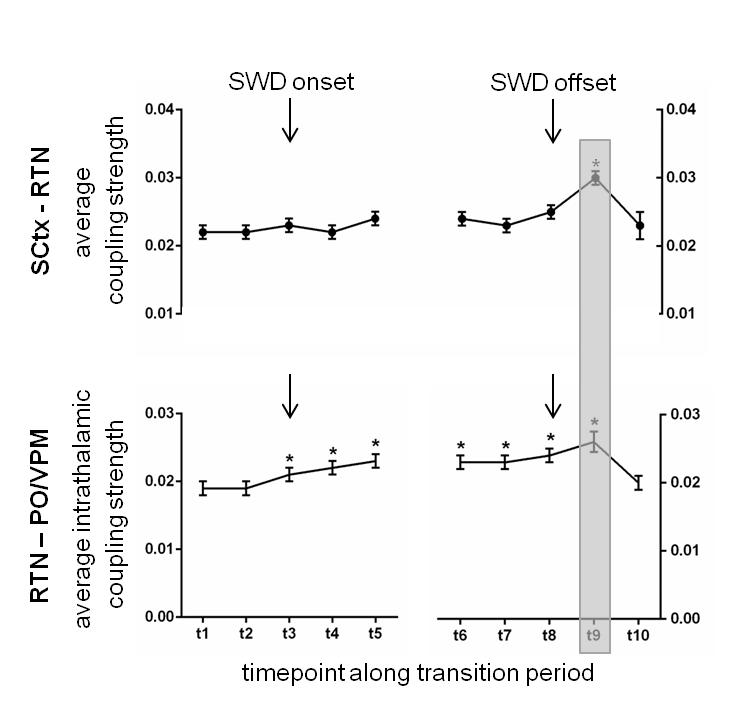 |
| --- |
| **Suppl. Fig. 4** Average intrathalamic coupling strength (i.e. coupling between RTN – VPM and RTN – PO) (lower panel) as compared to average coupling strength between SCtx and RTN (upper panel) represented for 10 time-intervals of interest. Note the gradual increase in intrathalamoc coupling strength during the SWD that reaches its maximum at time-interval t9 (i.e. immediately following SWD offset) coinciding with the strong short lasting increase in coupling strength between SCtx and RTN. |
